# Supplementary material for: Quantitative analysis of myofiber type composition in human and mouse skeletal muscles
Source: STAR Protoc. 2023 Jan 29;4(1):102075. doi: 10.1016/j.xpro.2023.102075 (PMC9898062; doi:10.1016/j.xpro.2023.102075)
Supplement: Document S1. Figure S1 [file mmc1.pdf]

STAR Protocols

CellPress
OPEN ACCESS

Protocol

Quantitative analysis of myofiber type composition in human and mouse skeletal muscles

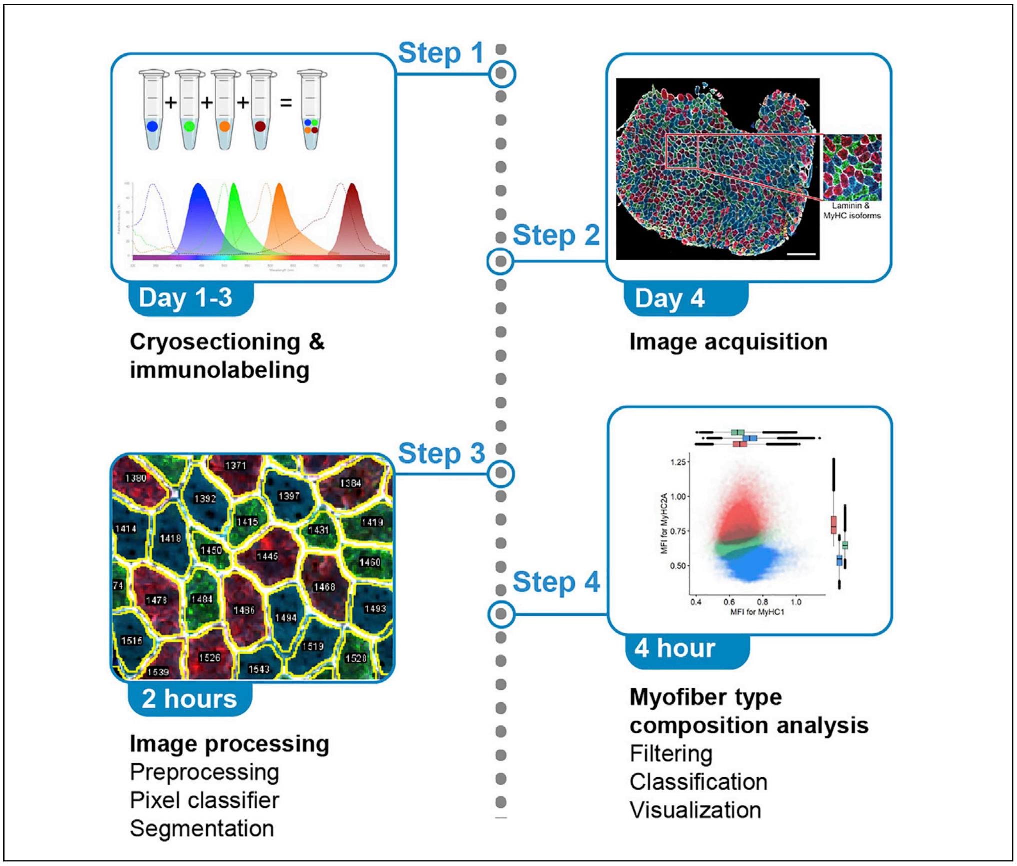

Skeletal muscles are composed of different myofiber types characterized by the expression of myosin heavy chain isoforms, which can be affected by physical activity, aging, and pathological conditions. Here, we present a step-by-step high-throughput semi-automated approach for performing myofiber type quantification of entire human or mouse muscle tissue sections, including immunofluorescence staining, image acquisition, processing, and quantification.

Publisher's note: Undertaking any experimental protocol requires adherence to local institutional guidelines for laboratory safety and ethics.

Tooba Abbassi-Daloii, Salma el Abdellaoui, Hermien E. Kan, Erik van den Akker, Peter A.C. 't Hoen, Vered Raz, Lenard M. Voortman

v.raz@lumc.nl (V.R.)
l.m.voortman@lumc.nl (L.M.V.)

Highlights

Image acquisition and image processing of the entire muscle section

High-throughput and semi-automated image quantification

Training a pixel classifier to improve the myofiber segmentation

Quantitative analysis to determine myofiber type composition

Abbassi-Daloii et al., STAR Protocols 4, 102075
March 17, 2023 © 2023 The Authors.
https://doi.org/10.1016/j.xpro.2023.102075

Check for updates

STAR Protocols

CellPress
OPEN ACCESS

Protocol

Quantitative analysis of myofiber type composition in human and mouse skeletal muscles

Tooba Abbassi-Daloii,[1] Salma el Abdellaoui,[1] Hermien E. Kan,[2,3] Erik van den Akker,[4,5,6] Peter A.C. 't Hoen,[1,7] Vered Raz,[1,9,*] and Lenard M. Voortman[8,9,10,*]

[1]Human Genetics, Leiden University Medical Center, 2333 ZA Leiden, the Netherlands

[2]C.J. Gorter MRI Center, Department of Radiology, Leiden University Medical Center, 2333 ZA Leiden, the Netherlands

[3]Duchenne Center Netherlands, the Netherlands

[4]Molecular Epidemiology, Leiden University Medical Center, 2333 ZC Leiden, the Netherlands

[5]Leiden Computational Biology Center, Leiden University Medical Center, 2333 ZC Leiden, the Netherlands

[6]Delft Bioinformatics Lab, TU Delft, 2628 XE Delft, the Netherlands

[7]Centre for Molecular and Biomolecular Informatics, Radboud Institute for Molecular Life Sciences, Radboud University Medical Center, 6525 GA Nijmegen, the Netherlands

[8]Cell and Chemical Biology, Leiden University Medical Center, 2333 ZA Leiden, the Netherlands

[9]Technical contact

[10]Lead contact

*Correspondence: v.raz@lumc.nl (V.R.), l.m.voortman@lumc.nl (L.M.V.)
https://doi.org/10.1016/j.xpro.2023.102075

SUMMARY

Skeletal muscles are composed of different myofiber types characterized by the expression of myosin heavy chain isoforms, which can be affected by physical activity, aging, and pathological conditions. Here, we present a step-by-step high-throughput semi-automated approach for performing myofiber type quantification of entire human or mouse muscle tissue sections, including immunofluorescence staining, image acquisition, processing, and quantification.

For complete details on the use and execution of this protocol, please refer to Abbassi-Daloii et al. (2022).[1]

BEFORE YOU BEGIN

There is a growing interest in the community to assess myofiber typing using multiplex staining[2] and image quantification,[3] which could result in robust analysis of changes in myofiber typing using machine learning.[4]

This protocol has been implemented for human and mouse muscles. Here, we explain the protocol using human samples. Furthermore, we show examples from mouse muscles and highlight the necessary adaptations in the protocol that should be made to optimize the method for a different species. We provide all the image processing macros and Rscripts on GitHub: https://github.com/tabbassidaloii/ImageProcessing/tree/main/MyofiberTyping.

Institutional permissions

Human muscle biopsies from healthy male subjects (aged 18–32) were used from a study detailed in Abbassi-Daloii et al.[1] The study was approved by the local Medical Ethical Review Board of The Hague Zuid-West and the Erasmus Medical Centre and conducted in accordance with the ethical standards stated in the 1964 Declaration of Helsinki and its later amendments (ABR number: NL54081.098.16). All subjects provided written informed consent prior to participation.

This is an open access article under the CC BY license (http://creativecommons.org/licenses/by/4.0/).

Table 1. Examples of fluorophore combinations for Axio Scan.Z1 slidescanner

	Different fluorophore combinations		
Conjugated antibody	Combination 1	Combination 2	Combination 3
MyHC1	Alexa Fluor® 350		
MyHC2A	Alexa Fluor® 594		
MyHC2B	Alexa Fluor® 488		
Primary + secondary antibody			
MyHC2X + anti-mouse secondary	Alexa Fluor® 488	Alexa Fluor® 488	Alexa Fluor® 647
Laminin + anti-rabbit secondary	Alexa Fluor® 647	Alexa Fluor® 750	Alexa Fluor® 750

Mouse muscle biopsies from eight-weeks-old male C57BL/6J wild-type mice were used from a study detailed in Bindellini et al.[5] Experiments in mice were approved and following the guidelines of the Animal Experiment Committee (DEC 13211) of the Leiden University Medical Centre.

Selecting the fluorophore combinations

Myofiber types are recognized with monoclonal antibodies to the three most abundant MyHC isoforms in humans: MyHC1 (clone #BA-D5), MyHC2A (clone #SC-71) and MyHC2X (clone #6H1). In mouse, MyHC2X was replaced with MyHC2B (clone #BF-F3). We made conjugated antibodies, allowing the detection of three MyHC isoforms and laminin in one staining. Antibody conjugation of #BA-D5, #SC-71, and #BF-F3 to Alexa Fluor 350, 594, and 488 fluorophores, respectively, is carried out with the antibody labeling kit (ThermoFisher), see key resources table for detailed information. The choice of Alexa Fluor fluorophores must consider which fluorescence channels are available in the imaging facility that is going to be used for the image acquisition. For each conjugated batch, the dilution factor should be determined on mouse or human muscle tissues. A rabbit-anti-laminin antibody, marking the cell boundary, is used to segment myofibers. The 6H1 and anti-laminin antibodies were detected with anti-mouse or anti-rabbit fluorescently conjugated secondary antibodies (Table 1). The choice for the fluorophore of the secondaries should be determined by the microscope filter combination, with the help of a spectra viewer tool (e.g., https://www.thermofisher.com/order/fluorescence-spectraviewer#!/). Examples of our possible choices to minimize spectral overlap are shown hereunder (Table 1 and Figure 1).

Note: We imaged the entire section with an automated slide scanning microscope (Zeiss Axio Scan.Z1). To determine which fluorophores to use, one should consider the imaging specifications of the microscope that is being used.

Testing the antibodies and determining the dilution factor is carried out on muscle cryosection using the immunofluorescence protocol detailed in section 1. Assessment of staining should be carried out with the same microscope that will be used for the entire experiment.

Preparing the antibody master mix

Prepare the master mix of the conjugated antibodies by appropriate dilution factor for the entire sections in an experiment to avoid the batch effect.

Note: The myofiber staining has many incubations and washing steps. Therefore, if carried out manually, we recommend including a maximum of 12 slides in each experimental batch.

Note: When there are multiple experimental batches, one should dilute the amount of antibodies required for the whole experiment in only one stock to avoid any variation. We advise testing diluted antibodies before running the large-scale experiment.

CellPress
OPEN ACCESS

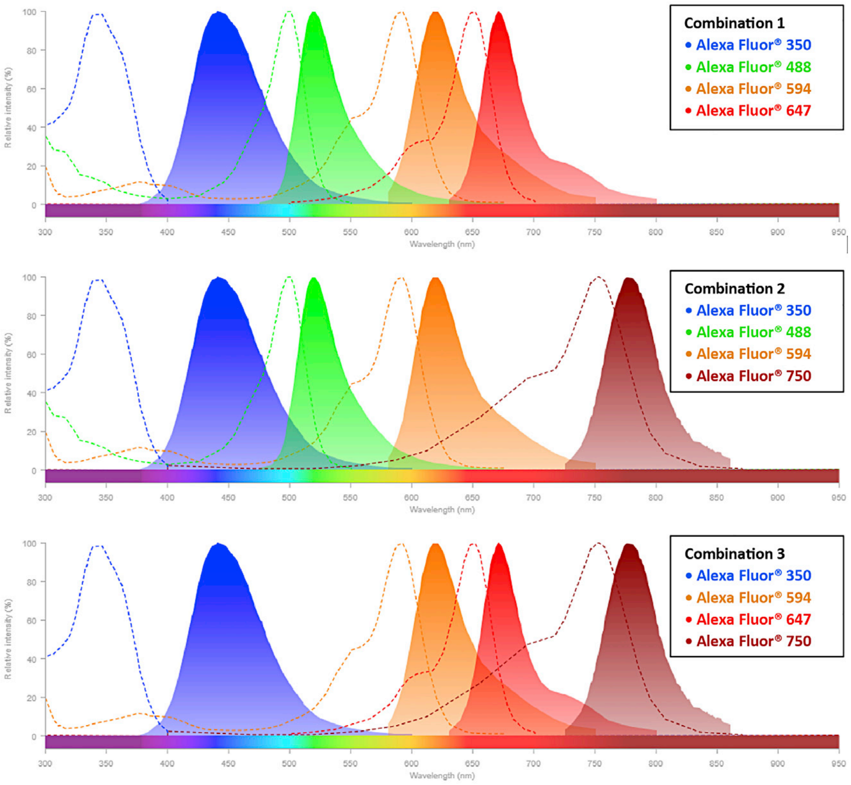

Figure 1. The spectra viewer for possible fluorophore combinations

Each graph represents the excitation spectra (dashed line) and emission spectra (solid line) of four different fluorophores specified in the boxes. These fluorophores have a minimal spectrum overlap showing their combination can be used for multi-channel imaging. *Note*: In this protocol, we use Alexa Fluor® 488 and Alexa Fluor® 750 as secondary antibodies for the myofiber staining.

KEY RESOURCES TABLE

REAGENT or RESOURCE	SOURCE	IDENTIFIER
Antibodies		
Myosin heavy chain type I antibody (1:600, conjugated with Alexa Fluor 350, to be determined per batch)	Developmental Studies Hybridoma Bank (DSHB)	Cat# BA-D5, RRID: AB_2235587
Myosin heavy chain type IIA antibody (1:700, conjugated with Alexa Fluor 594, to be determined per batch)	Developmental Studies Hybridoma Bank (DSHB)	Cat# SC-71, RRID: AB_2147165
Myosin heavy chain Type IIB antibody (1:600, conjugated with Alexa Fluor 488, to be determined per batch)	Developmental Studies Hybridoma Bank (DSHB)	Cat# BF-F3, RRID: AB_2266724
Myosin heavy chain, fast, 2X antibody (1:5)	Developmental Studies Hybridoma Bank (DSHB)	Cat# 6H1, RRID: AB_2314830
Anti-Laminin antibody produced in rabbit (1:1000)	Sigma-Aldrich	Cat# L9393
Goat anti-Mouse IgG (H+L) Cross-Adsorbed Secondary Antibody, Alexa Fluor™ 488 (1:1000)	Thermo Fisher Scientific	Cat# A11001, RRID: AB_2534069
Goat anti-Rabbit IgG (H+L) Cross-Adsorbed Secondary Antibody, Alexa Fluor™ 750 (1:1000)	Thermo Fisher Scientific	Cat# A-21039, RRID: AB_2535710
Alexa Fluor™ 350 Antibody Labeling Kit (see above)	Thermo Fisher Scientific	Cat# A20180
Alexa Fluor™ 488 Antibody Labeling Kit (see above)	Thermo Fisher Scientific	Cat# A20181
Alexa Fluor™ 594 Antibody Labeling Kit (see above)	Thermo Fisher Scientific	Cat# A20185
Chemicals, peptides, and recombinant proteins		
Ethanol 70%	N/A	N/A
OCT Embedding matrix for frozen sections (Tissue-Tek)	VWR, part of Avantor	Cat# 361603E

(*Continued on next page*)

Continued		
REAGENT or RESOURCE	SOURCE	IDENTIFIER
NaCl	Sigma-Aldrich	Cas# 7647-14-5
Na2HPO4.2H2O	Sigma-Aldrich	Cas# 10028-24-7
KCl	Sigma-Aldrich	Cas# 7447-40-7
KH2PO4	Sigma-Aldrich	Cas# 7778-77-0
Tween	Sigma-Aldrich	Lot# MKBK1089V
Milk powder	FrieslandCampina	
Software and algorithms		
ZEN Blue	Carl Ziess	https://www.zeiss.com/microscopy/int/products/microscope-software/zen/free-60-day-version-of-zen-blue-edition.html
ZEN Lite	Carl Ziess	https://www.zeiss.com/microscopy/int/products/microscope-software/zen-lite.html
Fiji	Schindelin et al.[6]	https://imagej.net/Fiji
Ilastic	Berg et al.[7]	https://www.ilastik.org/index.html
R	R-Core-Team[8]	https://www.r-project.org/
RStudio	RStudio-Team[9]	https://www.rstudio.com/
Other		
Cover slip	Menzel-Glaser	Lot #1180
ZEISS Axio Scan.Z1, Axioscan 7	Carl Ziess Microscopy GmbH	
Epredia™ SuperFrost™ Microscope Slides, Ground 90°	Thermo Fisher Scientific	Cat#12372098
Bright-field microscope	N/A	N/A
Immunopen	Dakocytomation	Cat#P36930,
Tweezers	N/A	N/A
Leica CM3050 S Cryostat	Leica Biosystems	N/A
Glass Insert 70 mm Wide For Anti-Roll Systems	Leica Biosystems	Cat#14047742497
Epredia™ MX35 Premier™ Disposable Low-profile Microtome Blades	Thermo Fisher Scientific	Cat#3052835

MATERIALS AND EQUIPMENT

Phosphate-buffered saline (PBS) 10×		
Reagent	Final concentration	Amount
NaCl	N/A	80 g
Na2HPO4.2H2O	N/A	15 g
KCl	N/A	2 g
KH2PO4	N/A	1,2 g
distilled water	N/A	up to 1,000 mL
**Total**	**10×**	**1,000 mL**

Phosphate-buffered saline containing 0.05% tween (PBST)		
Reagent	Final concentration	Amount
Tween	0.05%	0.5 mL
PBS	1×	999.5 mL
**Total**	**N/A**	**1,000 mL**
Store the PBS and PBST at 15°C–25°C up to 3–6 months.		

PBST containing 5% milk		
Reagent	Final concentration	Amount
Milk powder	5%	2.5 mg
PBST	1×	50 mL
Freshly made, can be stored at 4°C for one or two days.		

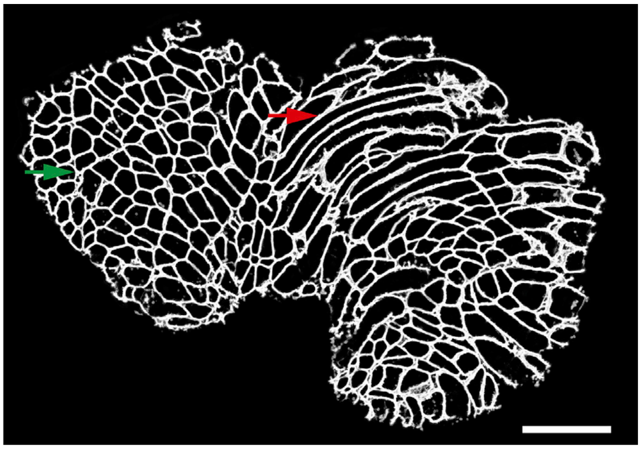

Figure 2. A representative image of muscle cryosection
The green arrow shows an area with cross-sections of myofibers, while the red arrow represents an area with longitudinal sections of myofibers. These regions with longitudinal sections are automatically excluded from the analyses later in the protocol. Scale bar 500 μm.

STEP-BY-STEP METHOD DETAILS

Cryosectioning of muscle biopsies

Timing: 20–30 min per muscle biopsy

The purpose of this step is to cryosection muscle biopsies for immunofluorescence staining.

Note: For users unfamiliar with cryosectioning, we recommend reading the article by Ross et al.,[10] which provides a detailed protocol.

Here, we describe the procedure for collecting cryosections:

1. Clean all the equipment with 70% alcohol.
2. Adjust the chamber temperature and the object temperature to −20°C and −22°C, respectively.
3. Place all the materials required for cutting (e.g., tweezers, specimen holder) inside the cryostat chamber to equilibrate to the temperature.
4. Install the anti-roll glass and a blade inside the cryostat chamber.
5. Adjust the thickness (we recommend cryosections of 16 μm thick for human tissue and 10 μm thick for mouse, but 8–16 μm thick leads to acceptable results).
6. Transport the muscle biopsy in liquid nitrogen to the cryostat.
7. Leave the biopsy inside the cryostat chamber to equilibrate to the temperature for 15–30 min.
8. Put some Tissue-Tek (depending on the biopsy size) on the specimen holder and place the muscle biopsy in the Tissue-Tek (using tweezers).
9. Place the specimen holder in the cryostat block when the muscle biopsy is completely fixed.
10. Collect the cryosections onto the SuperFrost slide.

Note: The tissue quality should be assessed by checking the first section under a normal bright-field microscope. Samples with extensive freezing damage should be excluded. In addition, since the purpose of this protocol is to perform myofiber typing for which cross-sections of myofibers are required, therefore you should examine if there are more cross-sections of myofibers rather than longitudinal sections of myofibers (Figure 2). If needed, the muscle

biopsy should be detached from the specimen holder and placed again with the correct orientation to get cross-sections.

11. Per biopsy, collect three cryosections onto SuperFrost slides.

Note: These three cryosections are used as technical replicates. Therefore, when pasting them onto a slide, make sure there is enough space around each cryosection to draw a circle using immunopen (step 15).

12. Store the slides at −20°C prior to staining.

Note: The slides can be stored at −20°C for several years.

Immunofluorescence

Timing: A day and a half per experimental batch

This section describes immunofluorescence staining using antibodies for three myosin heavy chain (MyHC) isoforms (MyHC1, MyHC2A, and MyHC2X) and laminin. Our protocol for immunofluorescence staining with the antibody mix of MyHC isoforms and laminin was described.[11]

Note: For conducting immunofluorescence staining in multiple experimental batches samples across groups should be randomized in different batches using a single antibody master mix. The antibody master mix can be stored at 4 degrees for 1–2 weeks.

Here, we provide step-by-step directions for conducting this experiment:

13. If slides were stored at −20°C, air dry them for 30 min at room temperature (RT). This incubation is important to limit detachment of the tissue during the incubations.
14. Outline each cryosection with an immunopen about 2–3 mm from the tissue edge.

Note: Do not draw the line too close to the muscle cryosections as it will introduce an artifact in the image processing step.

15. Wash the cryosections in PBST in the staining box.
16. Blocking.

Incubate slides in PBST + 5% milk for 30 min.

17. Wash the slides three consecutive times with a large volume of PBST, each time for 5 min.
18. Primary antibody incubation.
    Incubate sections with a mixture of the following primary antibodies for 2 h at RT:
    a. Rabbit anti-laminin,
    b. Mouse anti-6H1 detecting MyHC2X.

Note: The volume of the primary antibodies depends on the section area and you should make sure that the entire cryosection is covered with the antibodies.

19. Wash the slides three consecutive times with an excessive volume of PBST, each time for 5 min.

20. Secondary antibody incubation.
    Incubate sections with a mixture of the following secondary antibodies for 1 h at RT:
    a. Goat anti-rabbit-conjugated-Alexa Fluor® 750,
    b. Goat anti-mouse-conjugated-Alexa Fluor® 488.

Note: keep slides in the dark from step 21 onwards.

21. Wash the slides three consecutive times with an excessive volume of PBST, each time for 5 min.
22. Conjugated MyHC antibody mix incubation.
    Incubate the sections with a mixture of fluorescently conjugated monoclonal antibodies overnight at 4°C:
    a. BA-D5-conjugated-Alexa Fluor® 350, detecting MyHC1,
    b. SC-71-conjugated-Alexa Fluor® 594, detecting MyHC2A.

Note: Make sure that the sections don't dry out overnight. The slides can be carefully placed on wet tissue.

23. Wash the slides three consecutive times with an excessive volume of PBST for 5 min.
24. Wash the slides once with an excessive volume of PBS for 5 min.
25. Mounting.
    a. Cover the sections with ProLong™ Gold antifade reagent.
    b. Cover the slide with a cover slip.

Note: Avoid any air bubbles on the sections as they will affect the image acquisition.

    c. Fix the cover slip with nail polish.
26. Place the mounted sample on a flat, dry surface.
27. Incubate for 24 h at room temperature in the dark.
28. Store slides at 4°C prior to imaging.

Image acquisition

Timing: 10–15 min (per sample)

Here, we describe the imaging of the entire muscle sections using an Axio Scan.Z1 slidescanner (Carl Zeiss, Germany) image capturing using ZEN 2 (blue edition) software (the v2.6 was used in this protocol).

Alternatives: Other microscopes with high capacity image acquisition equipped with four fluorescence channels can be used.

Note: The imaging settings should be optimized on a test slide to specify the exposure time and intensity per fluorophore, as exposure time and focusing algorithm fade the fluorophore signal.

Note: We recommend using the channel with the highest signal-to-noise ratio (in this dataset the MyHC2A conjugated-Alexa Fluor® 594 channel) to define the focus plane.

Note: For each fluorophore/channel, the intensity and exposure time should be optimized to get the best signal-to-noise ratio, without bleaching the fluorophores.

29. Make images with a 10×/0.45 Plan-Apochromat objective lens (Figure 3).

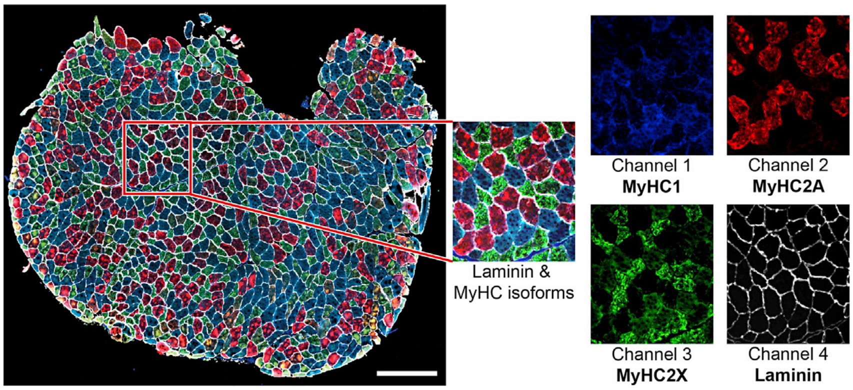

Figure 3. A representative immunostaining image
The left image shows the entire section, in the red box is a zoom-in image of the composite image and each myosin heavy chain isoform and laminin separately. Scale bar 500 μm.

30. Use single band filters for all channels:
    a. Channel 1 (MyHC1, Alexa Fluor® 350): 335 nm–383 nm excitation, 420 nm–470 nm emission in combination with 385 nm LED excitation wavelengths;
    b. Channel 2 (MyHC2A, Alexa Fluor® 594): 574 nm–599 nm excitation, 612 nm–682 nm emission in combination with 567 nm LED excitation wavelengths;
    c. Channel 3 (MyHC2X, Alexa Fluor® 488): 450 nm–490 nm excitation, 500 nm–550 nm emission in combination with 475 nm LED excitation wavelengths;
    d. Channel 4 (Laminin, Alexa Fluor® 750): 672 nm–747 nm excitation, 765 nm–855 nm emission in combination with 735 nm LED excitation wavelengths.

Note: The same image acquisition settings must be used for all slides over all batches.

Note: When you perform the staining in multiple batches, the imaging should be also done in the same order and batches to keep the same duration between staining and imaging for all batches.

Note: For each slide, the output is a Carl Zeiss Image format (CZI) dataset, which includes an image for each section.

Image preprocessing

Timing: 1–2 min (per sample)

Note: A video walkthrough of the image preprocessing and image processing steps described below can be found in Methods video S1.

Before the acquired images can be processed, the data needs to be curated, and some preprocessing might be required as explained below. The result of steps 32 and 33 should be a folder of multichannel tiff files with a pixel size between 1 and 5 μm that can be imported by Fiji.[6]

31. Calculating shading profile.
    If the acquired images exhibit a significant amount of shading (Figure 4A), this should be corrected since these intensity variations are not related to differences between distinct myofibers (Figure 3). The acquisition software offers a number of functions for shading correction: as an online process during the acquisition, or as a post-processing step.

CellPress
OPEN ACCESS

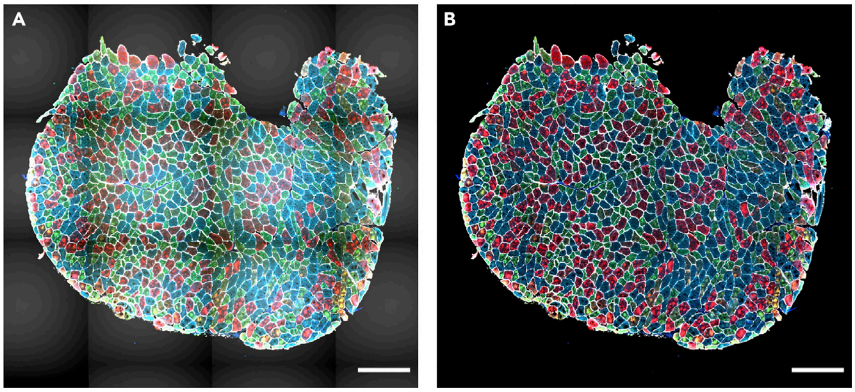

Figure 4. Shading correction
(A and B) A representative image before shading correction (A) and after shading correction (B). Scale bar 500 μm.

When these options do not give satisfactory results, it is possible to calculate 'averaged' shading profiles and use these for post-processing. When imaging with Axio Scan.Z1 slidescanner, this is achieved using ZEN Lite (v3.3 used for this protocol) and Fiji as below:

a. Calculate a shading profile using the 'Shading Reference From Tile Image' in ZEN Lite for each channel in each slide.
   i. This will produce a shading profile for each channel per slide.
b. Load all shading profiles for one specific channel into Fiji and combine them into a stack using the 'Images to stack' command.
c. Using 'Z Project' with 'Projection type' set to 'Median', calculate the median shading profile for this channel.
d. Repeat steps b-c for all channels.
e. Use the median shading profiles for the shading correction using 'Shading Correction' in ZEN Lite. This can only be done one channel at a time (Figure 4).

32. Converting image format.
Depending on the slide scanner used, this can be achieved in a multitude of ways. When using the Axio Scan.Z1 slidescanner the following procedure can be used in Fiji:
a. Open Fiji.
b. Run "0.Convert_CZI_to_Tiff.ijm" macro to convert the slidescanner datasets from CZI to multichannel 16-bit TIFF files using BioFormats.[12]
c. Provide a directory with CZI datasets.
   i. Running this macro, the images will be downsampled (4×) by averaging to improve the processing speed and reduce the required data storage.
   ii. The effective pixel size is 2.6 μm after downsampling.

Note: The effective pixel size will depend on the magnification of the acquisition. One should modify this to achieve a pixel size between 1 and 5 μm. It can be tuned by changing the scale parameter in "0.Convert_CZI_to_Tiff.ijm" macro (line 39). The amount of downscaling should be evaluated carefully by checking the output of the subsequent segmentation steps. If the segmentation quality is not sufficient, consider reducing the amount of downsampling.

d. For each image in each CZI dataset, a tiff file that ends with "_s[X]_merged" will be saved in the input directory provided.
   i. X shows the image number in the CZI dataset (starting from zero).

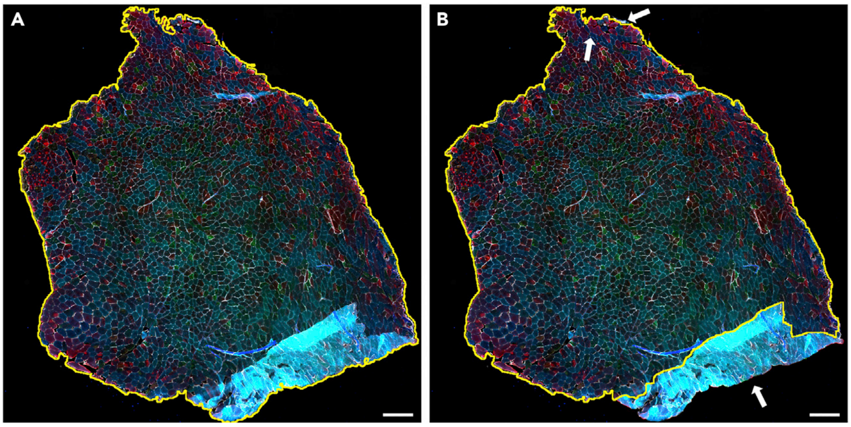

Figure 5. Tissue mask generation
(A) An automated tissue mask (thin yellow line).
(B) The adjusted mask excludes staining artifacts on the edges, out-of-focus regions, scratches, and dirt objects. Scale bar 500 μm.

Image processing

Timing: 5–10 min (per sample)

Image processing will be performed using Fiji and Ilastik[7] software.

Note: A modular set of macros that process each step independently is created. All the macros are publicly available on GitHub: https://github.com/tabbassidaloii/ImageProcessing/tree/main/MyofiberTyping/Macros. In the steps below, we specify which macro should be used.

Note: Macros save intermediate image files to facilitate debugging and rerunning certain steps when required.

33. Generating tissue mask.
This step is a semi-automated process that uses an automated mask generation algorithm, followed by a manual step to check and correct the generated masks.

Note: The aim of these manual corrections is to remove artifacts such as tissue folds, out-of-focus regions, scratches, and dirt objects.

Note: The image processing steps below expect a folder that contains multichannel 16 bit tiff files that have a filename that ends with "_merged.tif". These files should have an effective pixel size of approximately 2.6 μm.

a. Open Fiji.
b. Select Freehand Selection Tool.
c. Run "1.Tiff_to_Mask.ijm" macro to generate a mask for each image.
d. Provide a directory with merged tiff images.
   i. The first image will pop up with a mask generated automatically and a question box: "Is the mask OK?"
   ii. Press the OK button, if the generated mask is accurate. Otherwise, the mask can be adjusted (Figure 5):
   Zoom in or zoom out on the area of interest by holding Ctrl and scrolling the mouse wheel.

Hold Shift and Right Click on the area of the image that needed to be added to the mask;

Hold Alt and Right Click on the area of the image that needed to be removed from the mask.

Press OK when the mask is accurate to go to the next image.

Note: This manual process is the most time-consuming step in image processing.

e. For each image, a tiff file that ends with "_Mask" will be saved in the input directory provided.

34. Generating 'masked' copy of the laminin channel.
In this step, to reduce any possible artifacts due to this binary mask, a gaussian blur is applied to the mask and the pixel values of the laminin channel outside the mask are set to the median intensity of these pixels.

Note: We typically use a gaussian blur of 4 pixels (sigma = 4), but this can be tuned if the masks blur details too much (troubleshooting 1).

a. Open Fiji.
b. Run "2.Masked_Lamin.ijm" macro to generate a masked copy of laminin for each image.
c. Provide a directory with "_Mask" tiff images.
d. For each image, a tiff file that ends with "_Lamin_Masked" will be saved in the input directory provided.

35. Laminin segmentation using *Ilastik*.
In this step, to segment the myofibers, the masked laminin images will be fed into an *Ilastik*[7] pixel classification algorithm.

Note: To train this classifier only a small number of annotations on a small subset of images is required. After this training, the classifier can then be run on the entire dataset.

Note: For users unfamiliar with pixel classification in *Ilastik*, we recommend reading the *Ilastik* documentation here: https://www.ilastik.org/documentation/pixelclassification/pixelclassification.

a. Run *Ilastik* software.
b. Create a new "Pixel Classification" project.
c. Input data.
   i. Select "1. Input Data" menu on the left.
   ii. Click on "Add New" button to add a separate image(s).
   iii. Select multiple images (output of the previous step with "_Lamin_Masked" extension) that represent your dataset.
   iv. Click on "Open" button.
d. Feature selection.
   i. Select "2. Feature Selection" menu on the left.
   ii. Define features by clicking on "Select Features…" button as shown in Figure 6.
e. Training.
   i. Select "3. Training" menu on the left.
   ii. To train the classifier, define labels corresponding to two classes: 'myofiber boundary' and 'not myofiber boundary' (Figure 7).

Zoom in on different areas to annotate the classes.

Select one of the classes.

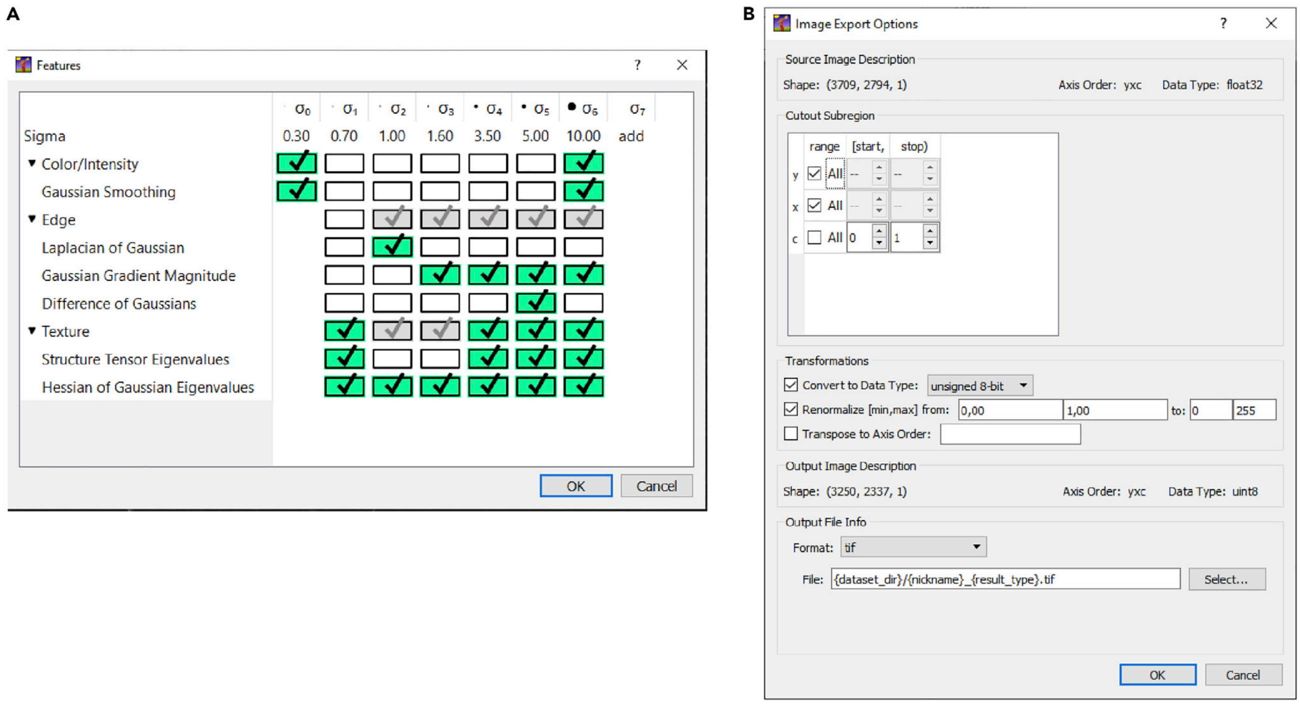

Figure 6. Feature selection and export settings in *Ilastik*

(A and B) The overview of features that should be selected (A) and the required settings for the Export Image Settings (B) for the pixel classification algorithm in *Ilastik*.

Select the pen with the proper size to annotate the pixels which belong to the class selected.

Select the other class and repeat step 3.

Evaluate the classifier performance by clicking on "Live Update" button and selecting "Segmentation" option (from "Group Visibility").

If required, improve the pixel annotation in each class.

Train and evaluate the classifier on at least three different input images by selecting them from "Current View".

Note: Annotate pixels in multiple areas of each section representing the tissue characteristics.

Note: Avoid overtraining the algorithm on a single section or area, as this will reduce the classifier performance across the whole dataset.

Note: When the classifier works as you expect, we recommend continuing with image processing and evaluating the performance of the classifier based on the output of the next step.

f. Prediction export.
   i. Select "4. Prediction Export" from the menu on the left.
   ii. Define the export image settings by clicking on "Choose Export Image Settings" as shown in Figure 7.
   iii. Save the project.

Note: An example of an *Ilastik* classifier file can be found on GitHub (https://github.com/tabbassidaloii/ImageProcessing/tree/main/MyofiberTyping/Macros) under the name of '3.Pixelclass_Lamin_Masked.ilp'.

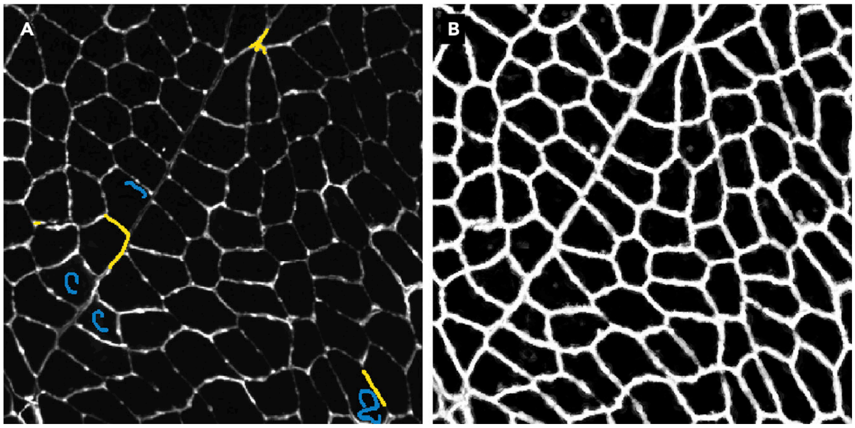

Figure 7. Training the classifier

(A) The annotation of the pixels which belong to the 'myofiber boundary' (yellow) and 'not myofiber boundary' (blue).
(B) result of the pixel classification showing the probability for each pixel belonging to the 'myofiber boundary' class (ranging from 0 in black, to 1 in white).

g. Run classifier.
   i. Use this classifier to process all images.
      Open Command Prompt.
      Run the command below by providing paths required:

```
``[Path to Ilastik]\ilastik.exe'' -headless -project="[Path to classifier saved]\3.pixel-
class_lamin_masked.ilp'' [Path to image]\*_Lamin_Masked.tif
```

      For each image, a tiff file that ends with ''Masked_Probabilities'' will be saved in the image directory.

36. Laminin segmentation and myofiber region-of-interest (ROI) generation.
    In this step, laminin segmentation is used to generate the regions-of-interest (ROI) (individual myofibers) for each image.
    a. Open Fiji.
    b. Run ''4.Segment_Lamin.ijm'' macro to segment and generate the ROIs for each image.
    c. Provide a directory with ''Masked_Probabilities'' tiff images.
    d. For each image.
       i. a tiff file (with ''_Segmentation'' extension) in the input directory provided.
          and,
       ii. an ROI file that ends with ''_ROI'' in the ROI subdirectory will be saved (Figure 8).
37. Extracting the mean-fluorescence-intensity and ROI properties.
    In this step, the mean-fluorescence-intensities (MFIs), as well as other properties in ROIs in all fluorescence channels, are extracted using the Fiji measurements: ''Area'', ''Mean gray value'', ''Standard deviation'', ''Modal gray value'', ''Min & max gray value'', ''Shape descriptors'', ''Median''.

Note: Macro, used in this step, adds an extra channel (channel 5) to show the results of the pixel-classification step. This 'classification' channel is the output of the pixel classification algorithm (step 36). The segmentation quality is evaluated using the ''Mean gray value'' on the border (strip of 3-pixels around ROI) of each ROI. This measurement allows assessment of the myofiber 'segmentation certainty' by looking at the 'classification' channel, i.e., the certainty

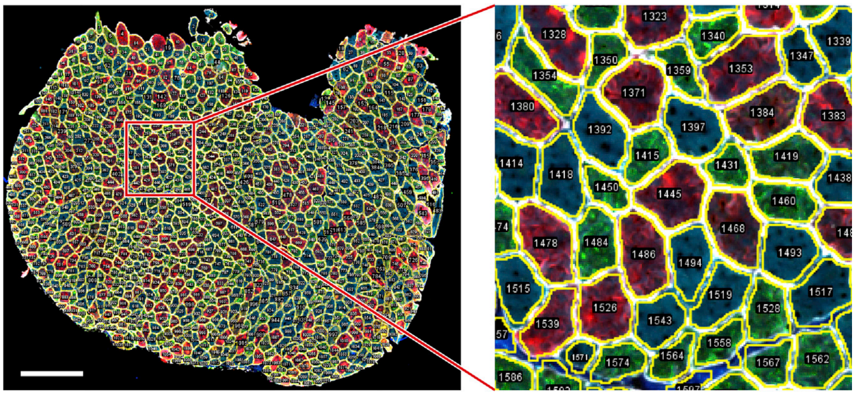

Figure 8. The ROI generation

A representative image of myofiber ROI segmentation. Scale bar 500 μm.

is high when the pixel-classification is high for the 'myofiber boundary' class all around the myofiber and low in the interior of the myofiber.

a. Open Fiji.
b. Run "5.Export_MFI_and_Laminin_Int_and_Distance.ijm" macro for the laminin segmentation and generate the regions-of-interest (ROI) (individual myofibers) for each image.
c. Provide a directory with tiff images.
d. For each image, a file and three images will be saved.
   i. a tab-delimited text file that ends with "_MFI" in the ROI subdirectory (Table 2).
   ii. three jpeg images that end with "_check[X]" in the check subdirectory.

Note: The distance of each myofiber to the edge of the tissue (Table 2) can be used to remove myofibers in case of suspected artifacts, such as close to the tissue borders (troubleshooting 2).

Note: To aid interpretation and verification of the results, visualizations of the segmentation, as well as the measured parameters such as mean fluorescence intensity are generated (jpeg images ending with "_check[X]").

Note: All the steps detailed above can be executed by running two Windows Batch Files provided on GitHub: https://github.com/tabbassidaloii/ImageProcessing/tree/main/MyofiberTyping/Macros/BatchFiles.

Myofiber type composition analysis

Timing: Half a day (depends on the dataset size)

In this section, we explain the filtering of non-myofiber ROIs and myofiber type classification. Here, we perform all the analyses in RStudio Software (v1.3.959 used for this protocol)[9] using R Statistical Software (v4.0.2 used for this protocol).[8] The R Markdown file is available on GitHub: https://github.com/tabbassidaloii/ImageProcessing/blob/main/MyofiberTyping/Rscript/MyofiberTyping.Rmd. In the steps below, we specify which R code chunk in this R markdown file should be used.

Note: The threshold used in the R markdown file is specific to one example dataset that was used in this protocol. The filtering threshold should be tuned for each dataset.

Table 2. An example of a tab-delimited file with the recorded measurements for one myofiber ROI

Label	Area	Mean	Circ.	Mean_boundary	StdDev_boundary	Mean_distance
0005-0074:1	6327.373	1903.1	0.86	1529.33	645.033	27.58
0005-0074:2	6327.373	213.77	0.86	195.31	39.079	27.58
0005-0074:3	6327.373	2151.1	0.86	1450.98	590.775	27.58
0005-0074:4	6327.373	1306.7	0.86	2330.43	760.089	27.58
0005-0074:5	6327.373	45.78	0.86	250.67	12.155	27.58

The Mean values for channels 1, 2 and 3 will be used to determine myofiber type in subsequent analysis. Channel 4 is not used in subsequent analysis. Channel 5 is the 'pixel classification score' that enables us to verify the quality of the segmentation. Please note that some values are duplicates (e.g., area and circularity only depend on the shape of the ROIs, and are therefore the same for all channels), and not all values are used for downstream analysis, these have been grayed out. **Label** consists of a unique identifier of the ROI and the channel number. **Area** is the cross-sectional area (CSA). **Mean** is the mean gray value intensity inside the object. **Circ.** is the circularity. **Mean_boundary** and **StdDev_boundary** are the mean gray value intensity and the standard deviation of the intensity on the object's boundary. **Mean_distance** is the object's mean distance to the edge of the section.

38. Exclusion of non-myofiber ROIs.
    In image processing, the laminin segmentation is automated, which may include non-myofiber ROIs in the dataset. Therefore, the first step is to filter out the non-myofiber ROIs. We consider percentiles of 'segmentation certainty', CSA, and circularity values.

Note: Filtering out using a statistical value could result in removing true myofibers ROIs. However, due to the large myofiber ROIs, the dataset outcome is unaffected by small changes in percentile cutoff values.

Note: We verify the thresholds applied by visualizing the filtered ROIs using "6.Visual_Check_Filtering.ijm" macro available on GitHub: https://github.com/tabbassidaloii/ImageProcessing/tree/main/MyofiberTyping/Macros. The visualization can be performed after each filtering step to justify the threshold. Here we only show an example of filtered myofibers after the last filtering step.

a. Filtering based on segmentation certainty.
   The measurement in the 'classification' channel can be used to assess the segmentation certainty and filter out non-myofiber ROIs (Table 2).

Note: The measurements which are based on the "Mean gray value" are: *Mean*, laminin intensity inside the object (the smaller the better); *Mean_boundary*, laminin intensity on the boundary (the larger the better); *StdDev_boundary*, standard deviation of laminin intensity on the boundary (the smaller the better).

i. Pool all the data from all the samples by running "readDataset" R code chunk.
ii. Draw a density plot for each metric to define a filtering threshold by running "Filt1_segmentationMetrics_denPlot" R code chunk.
iii. Define the threshold based on the density distribution and apply filtering for Mean and Mean_boundary by running "Filt1_segmentationMetrics_filtering" R chunk code.
    In our dataset, including 369073 ROIs, we excluded ROIs from the top and bottom 5th percentile (Figure 9).

Note: In this example, filtering based on StdDev_boundary is not used, because the StdDev_-boundary has been improved by filtering for two other metrics.

Note: The filtering thresholds should be tuned for other input datasets. We recommend iteratively assessing the thresholds applied by visualizing the filtered ROIs.

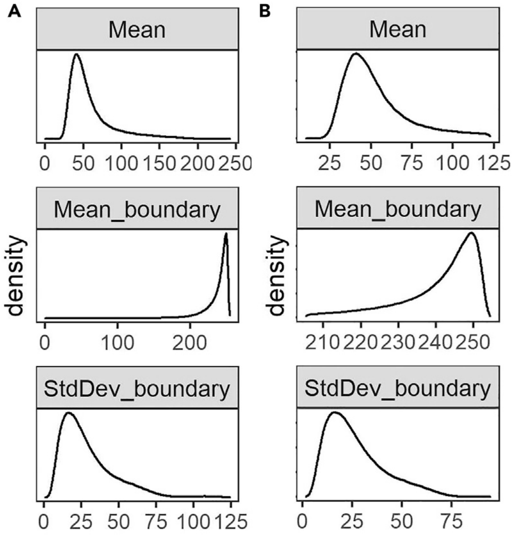

Figure 9. The distribution of three measurements defined to assess segmentation certainty

(A and B) The density plots of Mean, Mean_boundary, and StdDev_boundary across all the samples before (panel A) and after filtering (panel B).

b. Filtering based on CSA (μm2).
   The next filtering step uses the CSA: large CSA values could represent multiple myofibers that were not individually segmented due to the absence of laminin staining or small CSA values might be either small non-myofiber cells or a space between contiguous myofibers.
   i. Draw a density plot to define a filtering threshold by running "Filt2_CSA_denPlot" R code chunk (Figure 10A).
   ii. Apply filtering for CSA by running "Filt2_CSA_filtering" R chunk code.

Note: Include all ROIs including those that are filtered in the segmentation certainty filtering step.

Note: Depending on the dataset characteristic, CSA could have a diverse distribution across sample groups. Therefore, when the distribution is not the same, the filtering based on CSA should be separately applied to each group. However, applying filtering separately may introduce a bias, therefore these decisions about the study design require careful consideration. This is an important consideration to preserve biological differences between sample groups. We show an example for CSA across different muscles, we notice that the mean CSA differed between muscles, therefore the CSA-based filtering was made per muscle group.

Note: When the normal CSA range for a given sample group is known, filtering for CSA can be implemented by removing ROIs out of range.

Note: Since there is a right-skewed distribution, one may use different thresholds for both sides of the distribution (Figure 10B).

Note: Verify the thresholds can be made by visualization of the filtered ROIs using the "6.Visual_Check_Filtering.ijm" macro available on GitHub.

c. Filtering based on circularity.
   The circularity is an ROI measure, which ranges between 0 (elongated shape) and 1 (circle). This measure is used to filter out longitudinally sectioned myofibers and elongated ROIs that may represent multiple myofibers (mis-segmented ROIs).
   i. Draw a density plot of the entire dataset to define a filtering threshold by running "Filt3_circularity_denPlot" R code chunk (Figure 11A).

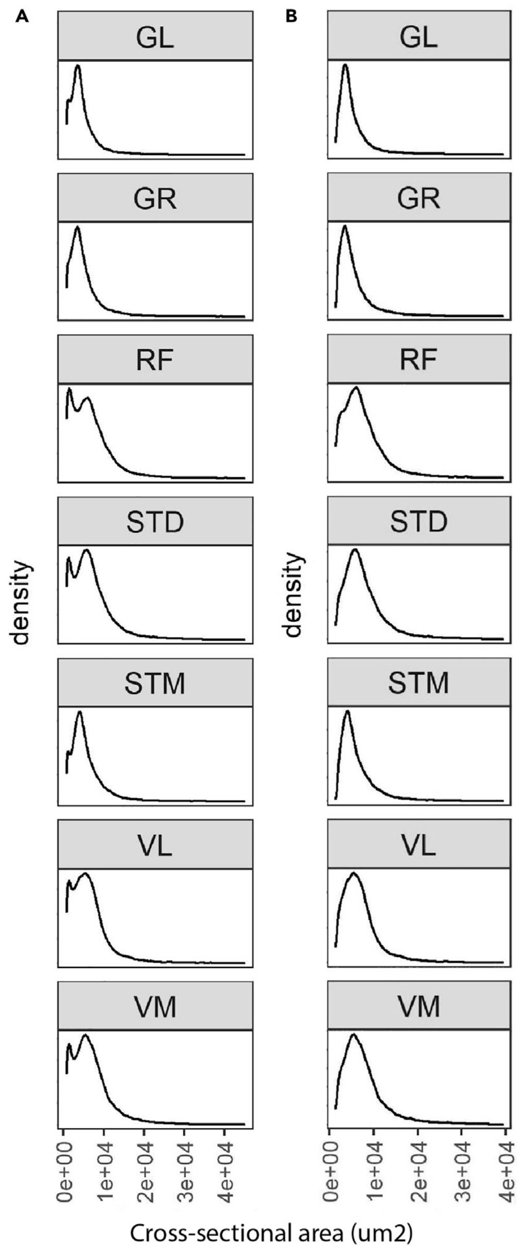

Figure 10. The CSA distribution in each muscle

(A and B) The CSA density plot before (A) and after (B) filtering. Here, we include ROIs with 10th percentile < CSA < 99th percentile. In this protocol, we used human samples collected from six leg muscles, gracilis (GR), semitendinosus (ST), rectus femoris (RF), vastus lateralis (VL), vastus medialis (VM), and gastrocnemius lateralis (GL) muscles. We also included biopsies from the middle (STD) and distal (STM) end of the semitendinosus muscle to investigate differences within one muscle. For this dataset, we tested different thresholds. Visualizing the myofibers excluded, we realized that if we exclude more than 1% on the right side, we would filter out 'real' myofibers (visual base). The small non-myofiber ROIs are not entirely excluded, but if we exclude more than 10% from the left (small ROIs), we would also filter out 'real' myofibers.

ii. Apply filtering for circularity by running "Filt3_circularity_filtering" R chunk code.
Based on density distribution, we include ROIs with Circularity > 1st percentile (Figure 11B).

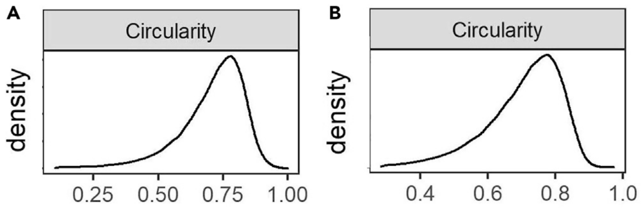

Figure 11. The circularity distribution in each muscle

(A and B) The circularity density plot before (A) and after (B) filtering.

Note: Apply filtering based on the percentile of all ROIs including those that are filtered in the previous filtering steps.

39. Visualizing and justifying the thresholds.
    In this step, we perform a spatial assessment of the excluded and included myofibers by visualizing the filtered ROIs using the "6.Visual_Check_Filtering.ijm" macro available on GitHub.
    a. Save a tab-delimited text file (with "_Filt" extension) in the ROI subdirectory by running "save_visualizationInput" R chunk code.
       In each filtering step, the script adds a column with 0 and 1 values to the tab-delimited text file specifying which ROIs are included (1) or excluded (0). The script also adds an extra column with values between 0 and 1 to show the aggregated filtering results. These values will be used to give a distinct color to ROI excluded in each filtering step:
       i. **0**: ROIs filtered based on segmentation certainty.
       ii. **0.4**: ROIs included after filtering based on laminin segmentation certainty.
       iii. **0.7**: ROIs included after filtering based on CSA.
       iv. **1**: ROIs included after filtering based on circularity.
    b. Open Fiji.
    c. Run "6.Visual_Check_Filtering.ijm" macro to visualize the filtered ROIs.
    d. Provide directory with tiff images and ROI subdirectory.
    e. For each sample, a jpeg file that ends with "_check4" will be saved in the image directory (Examples are in Figure 12).
40. Selecting one replicate per sample.
    In this step, one section per sample is selected for further analysis. We recommend selecting the section with the highest number of myofibers after all filtering steps considering the quality of the sections. We consider only samples with a minimum of one hundred myofibers for downstream analysis.
41. classification of myofiber types.
    The protocol for data-driven myofiber classification is detailed in Raz et al.[13] Myofiber classification, depending on the specific bandwidth, could result in 3–8 clusters, encompassing single MyHC myofiber type, hybrids, and potentially a cluster with all three MyHC isoforms and a cluster with only low MyHC intensity.
    a. Scaling and transformation.
       The MFI values for each of the three MyHC isoforms are scaled for each myofiber per sample (without centering) using "ScalingAndTransformation" R chunk code.
    b. Clustering.
       This step implements the mean-shift algorithm, a density-based clustering approach, implemented in the LPCM R package (v0.46-7)[14,15] to cluster the objects based on the transformed (natural logarithm) MFI values. The transformed scaled values for each MyHC isoform are used for clustering.
       i. Cluster the myofibers by running "clustering" R chunk code.

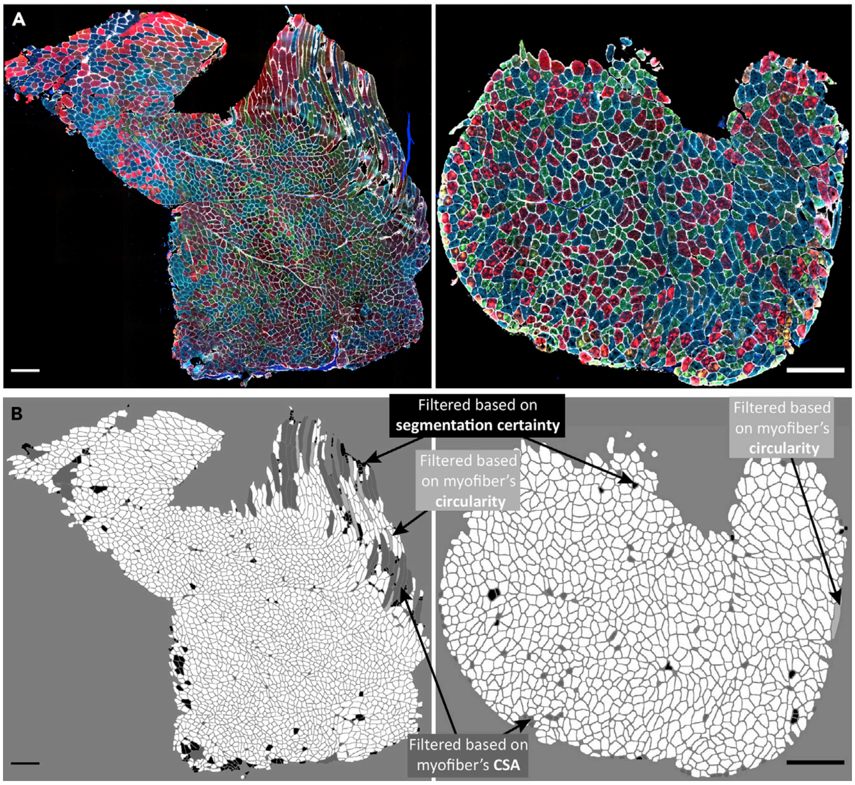

Figure 12. Visualization of myofiber filtering
(A) Immunostained images of two muscle sections.
(B) Visualization of excluded myofibers after filtering for segmentation certainty (black), CSA (dark gray), and circularity (light gray). Scale bar 500 μm.

Note: The optimum bandwidth (*h*) is arbitrary and should be selected based on the dataset characteristic. We suggest using *h* values ranging from 0.01 to 0.05 to find the optimum value. In general, a lower value assigns myofibers to many small clusters, whereas a higher value would result in grouping the myofibers in larger clusters. We expect to have three to eight clusters as potentially biologically relevant.

ii. Remove the cluster with a low proportion of myofibers (typically < ~2%).
iii. Draw boxplots to visualize the myofibers clusters (an example is in Figure 13).
iv. Generate a spatial visualization of the clustering assignment per myofiber (an example is in Figure 14).

42. Biological interpretation.

The proportion of myofibers in each cluster can be then calculated per sample and can be used to compare myofiber type composition.

Note: The expression of MyHC isoforms describes contraction capacity. Changes in myofiber type composition were reported in pathological and physiological conditions. A quantitative assessment of myofiber type composition can help in understanding disease progression,[13] and of a therapeutic assessment.[16] The biological relevance of myofiber type composition clusters is not fully exploited. However, it allows recognizing hybrids, which are implicated in aging.[13] Myofiber type clusters differ between muscle groups in both mouse and human,[1,17,18] although not fully understood, it could suggest a relevance for muscle physiology and function.

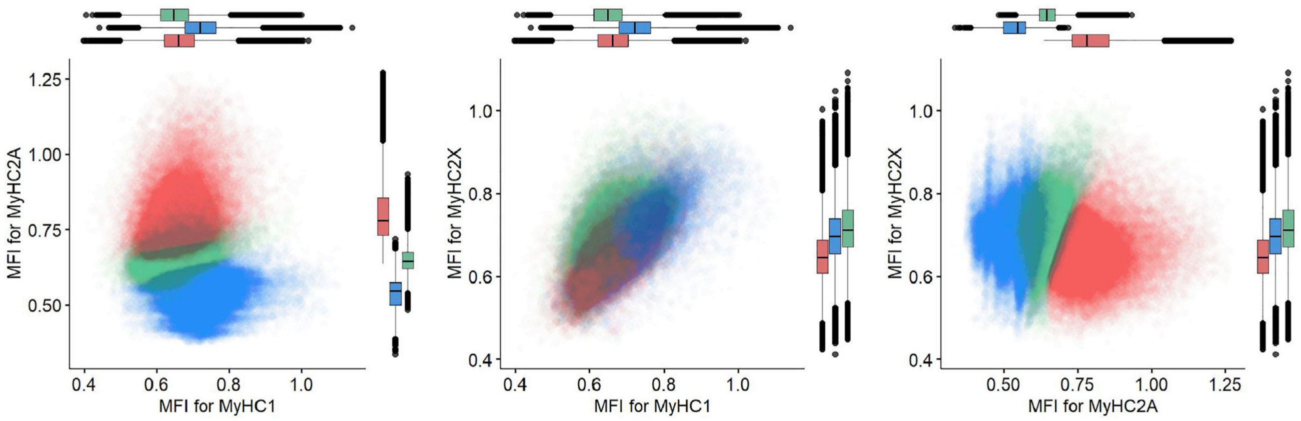

Figure 13. Myofiber types clustering example

Each dot represents a myofiber. In the dataset used for this protocol, we found three main clusters. Each myofiber cluster has a major MyHC isoform: MyHC2A is the major isoform in Cluster 1 (red), MyHC1 is the major isoform in cluster 2 (blue) and MyHC2X is the major isoform in cluster 3 (green). Boxplots show the MFI of the corresponding MyHC isoforms in three different clusters.

EXPECTED OUTCOMES

This protocol allows segmentation of the whole muscle section, quantification of the three main MyHC isoforms, and myofiber typing using a semi-automated pipeline. The main user interaction is to check and potentially correct automatically generated tissue masks to exclude tissue folds or other potential artifacts from the analysis. Even though the pipeline is highly automated, each individual step can be inspected to provide additional insight and to alleviate any potential issues. The main outcome of the image processing steps is a text file with the mean fluorescent intensity of these isoforms as well as other properties of each individual segmented myofiber including cross-sectional area and circularity. The file also includes the measurement for the 'classification' channel allowing assessment of the myofiber 'segmentation certainty'. In the myofiber type composition analysis steps, following the filtering of non-myofiber ROIs, the MFI of three MyHC is used to cluster myofibers and to calculate the myofiber type composition in each sample.

QUANTIFICATION AND STATISTICAL ANALYSIS

In this protocol we show how to use text files to calculate and compare the myofiber type compositions between different sample groups in R. But other parameters (e.g., cross-sectional area) can also be used for further comparison and statistical analysis. In addition, the text file can be imported into any other data analysis software such as Python or Excel.

LIMITATIONS

The laminin segmentation is performed in an automated manner because manual segmentation is nearly undoable for whole tissue sections. Some mis-segmentation is unavoidable, but the majority of mis-segmentations are removed using the filter procedures described above.

In addition, we only applied this protocol on human and mouse samples (see Figure S1), and one may need to optimize it for other species. Finally, our image processing and quantification steps may need to be adjusted for other imaging equipment and image analysis software.

TROUBLESHOOTING

The authors are available for troubleshooting and advice.

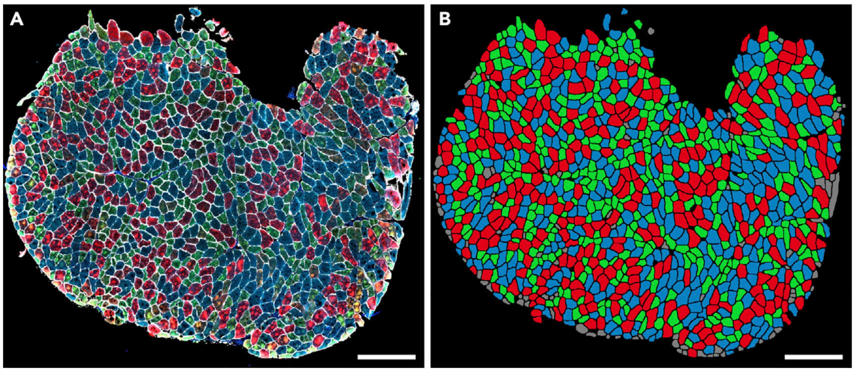

Figure 14. Visualization of myofiber clusters
(A) Immunostained images of a muscle section.
(B) Myofibers assigned to Cluster 1, Cluster 2, and Cluster 3 are depicted in red, blue, and green, respectively. Myofibers assigned to the small clusters or filtered in the filtering steps are shown in gray. Panel B was generated by visualization of the clustering result using the ImageJ macro "6.Visual_Check_Filtering.ijm", followed by a colorization step using image editing software. Scale bar 500 μm.

Problem 1

When applying a gaussian blur to reduce any possible artifacts due to this binary mask, some biological signals might be removed.

Potential solution

In this protocol, we use a gaussian blur of 4 pixels (sigma = 4), but if it removes too many details the sigma can be adjusted in the "2.Masked_Lamin.ijm" macro (line 46).

Problem 2

After Immunostaining, there might be more staining artifacts on the edge of the tissue.

Potential solution

We provide a measure showing the distance of each segmented myofiber to the edge of the tissue in the text output file. This measurement can be used to filter out myofibers with staining artifacts close to the tissue borders.

RESOURCE AVAILABILITY

Users can report any issues with running this protocol to https://github.com/tabbassidaloii/ImageProcessing/issues.

Lead contact

Further information and requests for resources and reagents should be directed to and will be fulfilled by the lead contact Lenard M. Voortman (l.m.voortman@lumc.nl).

Materials availability

This study did not generate new unique reagents.

Data and code availability

A small subset of the dataset generated in the current study has been deposited on *figshare*: https://doi.org/10.6084/m9.figshare.21324072.v2. All the image processing macros and R scripts are provided on GitHub: https://github.com/tabbassidaloii/ImageProcessing/tree/main/MyofiberTyping (https://doi.org/10.5281/zenodo.7466595).

SUPPLEMENTAL INFORMATION

Supplemental information can be found online at https://doi.org/10.1016/j.xpro.2023.102075.

ACKNOWLEDGMENTS

This project was funded by the Netherlands Organization for Scientific Research (NWO, under research program VIDI, grant # 917.164.90) and the Association Francaise contre les Myopathies (AFM Telethon; grant # 22506).

AUTHOR CONTRIBUTIONS

Conceptualization, T.A.D., L.V., V.R.; methodology, T.A.D., L.V., V.R.; software, T.A.D., L.V.; validation, T.A.D., L.V.; formal analysis, T.A.D., L.V.; investigation, T.A.D., S.E.A., L.V.; data curation, T.A.D., L.V.; writing – original draft, T.A.D., L.V.; writing – review & editing, T.A.D., S.E.A., P.A.C.H., H.E.K., V.R., L.V.; visualization, T.A.D., L.V.; supervision, T.A.D., L.V., V.R.; project administration, V.R., H.E.K.; funding acquisition, V.R., H.E.K.

DECLARATION OF INTERESTS

H.E.K. reports research support from Philips Healthcare and trial support from ImagingDMD. No personal fees are received, and all revenues go to the LUMC.

REFERENCES

1. Abbassi-Daloii, T., el Abdellaoui, S., Voortman, L.M., Veeger, T., Cats, D., Mei, H., Meuffels, D.E., van Arkel, E., 't Hoen, P.A.C., Kan, H.E., and Raz, V. (2022). A transcriptome atlas of leg muscles from healthy human volunteers reveals molecular and cellular signatures associated with muscle location. Preprint at bioRxiv. https://doi.org/10.1101/2022.06.01.494335.

2. Rao, V.V., and Mohanty, A. (2019). Immunohistochemical identification of muscle fiber types in mice tibialis anterior sections. Bio. Protoc. *9*, e3400. https://doi.org/10.21769/BioProtoc.3400.

3. Waisman, A., Norris, A.M., Elías Costa, M., and Kopinke, D. (2021). Automatic and unbiased segmentation and quantification of myofibers in skeletal muscle. Sci. Rep. *11*, 11793. https://doi.org/10.1038/s41598-021-91191-6.

4. Kastenschmidt, J.M., Ellefsen, K.L., Mannaa, A.H., Giebel, J.J., Yahia, R., Ayer, R.E., Pham, P., Rios, R., Vetrone, S.A., Mozaffar, T., and Villalta, S.A. (2019). QuantiMus: a machine learning-based approach for high precision analysis of skeletal muscle morphology. Front. Physiol. *10*, 1416. https://doi.org/10.3389/fphys.2019.01416.

5. Bindellini, D., Voortman, L.M., Olie, C.S., van Putten, M., van den Akker, E., and Raz, V. (2021). Discovering fiber type architecture over the entire muscle using data-driven analysis. Cytom. A *99*, 1240–1249. https://doi.org/10.1002/cyto.a.24465.

6. Schindelin, J., Arganda-Carreras, I., Frise, E., Kaynig, V., Longair, M., Pietzsch, T., Preibisch, S., Rueden, C., Saalfeld, S., Schmid, B., et al. (2012). Fiji: an open-source platform for biological-image analysis. Nat. Methods *9*, 676–682. https://doi.org/10.1038/nmeth.2019.

7. Berg, S., Kutra, D., Kroeger, T., Straehle, C.N., Kausler, B.X., Haubold, C., Schiegg, M., Ales, J., Beier, T., Rudy, M., et al. (2019). ilastik: interactive machine learning for (bio)image analysis. Nat. Methods *16*, 1226–1232. https://doi.org/10.1038/s41592-019-0582-9.

8. R-Core-Team. (2020). R: A Language and Environment for Statistical Computing (R Core Team).

9. RStudio-Team (2020). RStudio: Integrated Development Environment for R (RStudio Team).

10. Ross, M.A., Kohut, L., and Loughran, P.A. (2022). Cryosectioning. Curr. Protoc. *2*, e342. https://doi.org/10.1002/cpz1.342.

11. Riaz, M., Raz, Y., van Putten, M., Paniagua-Soriano, G., Krom, Y.D., Florea, B.I., and Raz, V. (2016). PABPN1-Dependent mRNA processing induces muscle wasting. PLoS Genet. *12*, e1006031. https://doi.org/10.1371/journal.pgen.1006031.

12. Linkert, M., Rueden, C.T., Allan, C., Burel, J.M., Moore, W., Patterson, A., Loranger, B., Moore, J., Neves, C., Macdonald, D., et al. (2010). Metadata matters: access to image data in the real world. J. Cell Biol. *189*, 777–782. https://doi.org/10.1083/jcb.201004104.

13. Raz, Y., van den Akker, E.B., Roest, T., Riaz, M., van de Rest, O., Suchiman, H.E.D., Lakenberg, N., Stassen, S.A., van Putten, M., Feskens, E.J.M., et al. (2020). A data-driven methodology reveals novel myofiber clusters in older human muscles. FASEB J. *34*, 5525–5537. https://doi.org/10.1096/fj.201902350R.

14. Cheng, Y. (1995). Mean shift, mode seeking, and clustering. IEEE Trans. Pattern Anal. Mach. Intell. *17*, 790–799. https://doi.org/10.1109/34.400568.

15. Einbeck, J. (2011). Bandwidth selection for mean-shift based unsupervised learning techniques: a unified approach via self-coverage. J. Pattern Recogn. Res. *6*, 175–192. https://doi.org/10.13176/11.288.

16. Olie, C.S., Riaz, M., Konietzny, R., Charles, P.D., Pinto-Fernandez, A., Kiełbasa, S.M., Aartsma-Rus, A., Goeman, J.J., Kessler, B.M., and Raz, V. (2019). Deacetylation inhibition reverses PABPN1-dependent muscle wasting. iScience *12*, 318–332. https://doi.org/10.1016/j.isci.2019.01.024.

17. Raz, V., Riaz, M., Tatum, Z., Kielbasa, S.M., and 't Hoen, P.A.C. (2018). The distinct transcriptomes of slow and fast adult muscles are delineated by noncoding RNAs. FASEB J. *32*, 1579–1590. https://doi.org/10.1096/fj.201700861R.

18. Raz, Y., Henseler, J.F., Kolk, A., Riaz, M., van der Zwaal, P., Nagels, J., Nelissen, R.G.H.H., and Raz, V. (2015). Patterns of age-associated degeneration differ in shoulder muscles. Front. Aging Neurosci. *7*, 236. https://doi.org/10.3389/fnagi.2015.00236.

STAR Protocols, Volume *4*

Supplemental information

Quantitative analysis of myofiber type composition in human and mouse skeletal muscles

Tooba Abbassi-Daloii, Salma el Abdellaoui, Hermien E. Kan, Erik van den Akker, Peter A.C. 't Hoen, Vered Raz, and Lenard M. Voortman

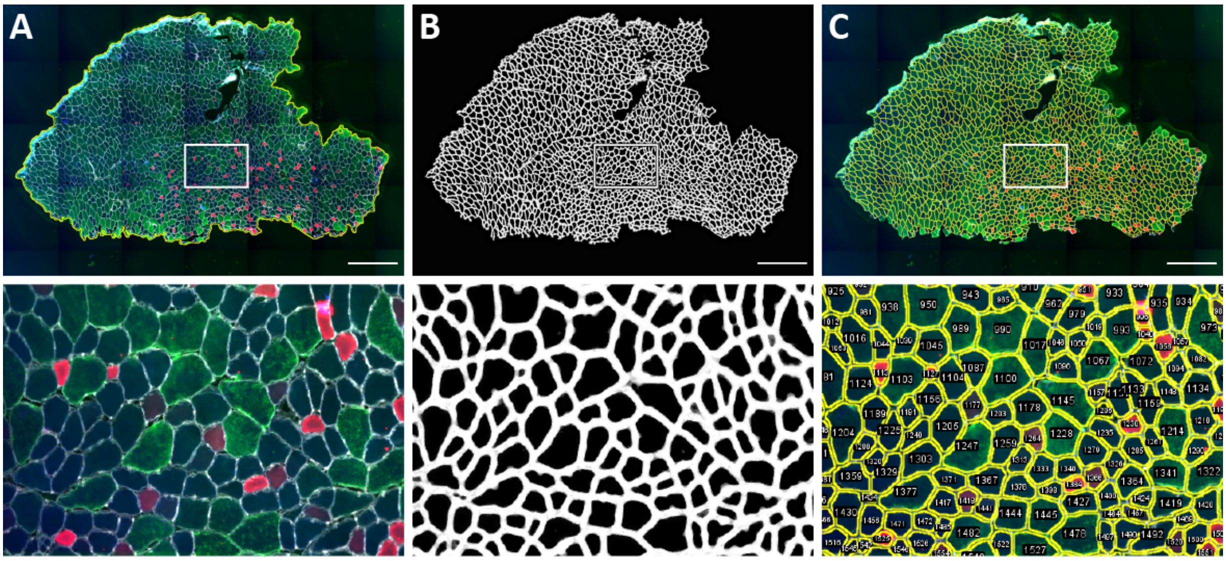

Supplementary Figure 1. Segmentation of mouse muscle section. A demonstration of the segmentation of mouse muscle. **A)** Equivalent to human muscle sections, the entire section is imaged. Thin yellow line is the automatically generated mask. **B)** Result of the Ilastik pixel classification. **C)** Resulting segmentation.
